# Research on Flow Field Perception Based on Artificial Lateral Line Sensor System

**DOI:** 10.3390/s18030838

**Published:** 2018-03-11

**Authors:** Guijie Liu, Mengmeng Wang, Anyi Wang, Shirui Wang, Tingting Yang, Reza Malekian, Zhixiong Li

**Affiliations:** 1 Department of Mechanical and Electrical Engineering & Key Laboratory of Ocean Engineering of Shang Dong Province, Ocean University of China, Qingdao 266100, China; wmm@stu.ouc.edu.cn (M.W.); wanganyi@stu.ouc.edu.cn (A.W.); wsr@stu.ouc.edu.cn (S.W.); yangtingting@stu.ouc.edu.cn (T.Y.); 2 Department of Electrical, Electronic & Computer Engineering, University of Pretoria, Pretoria 0002, South Africa; reza.malekian@ieee.org; 3 School of Mechanical, Materials, Mechatronic and Biomedical Engineering, University of Wollongong, Wollongong 2522, NSW, Australia; zhixiong.li@ieee.org

**Keywords:** artificial lateral line system, hydrodynamic simulation, flow field perception, velocity estimation, neural network

## Abstract

In nature, the lateral line of fish is a peculiar and important organ for sensing the surrounding hydrodynamic environment, preying, escaping from predators and schooling. In this paper, by imitating the mechanism of fish lateral canal neuromasts, we developed an artificial lateral line system composed of micro-pressure sensors. Through hydrodynamic simulations, an optimized sensor structure was obtained and the pressure distribution models of the lateral surface were established in uniform flow and turbulent flow. Carrying out the corresponding underwater experiment, the validity of the numerical simulation method is verified by the comparison between the experimental data and the simulation results. In addition, a variety of effective research methods are proposed and validated for the flow velocity estimation and attitude perception in turbulent flow, respectively and the shape recognition of obstacles is realized by the neural network algorithm.

## 1. Introduction

Most fishes are dependent on the lateral organs to detect their surrounding environment, track moving targets and avoid obstacles [1]. In steady flow, they rely on lateral sensing information to estimate flow velocity [2], direction [3], realize rheotaxis and reduce energy consumption in different swimming state [4]. So, the function of the fish lateral-line provides a new idea for underwater perception.

At present, many researchers have carried out detailed studies about artificial lateral-line sensor’s design, fabrication and optimization [5]. They have developed different sensing systems, conducted parametric underwater experiments and established related algorithms [6]. Pandya produced an artificial lateral-line system equipped with commercial hot-wire anemometer sensors and micro-mechanical sensors, which successfully located a moving dipole source [7]. Venturelli developed a rigid parallel distributed lateral line system installed with 20 pressure sensors, which achieved the purpose of fluid environment identification and Karman vortex streets detection [8]. Chambers produced a three-dimensional fish head with 33 pressure sensors (MS5401-AM) to study the fluid interaction [9]. Levi DeVries designed an artificial system based on 8 IPMC sensors and 4 pressure sensors, which estimates flow parameters for feedback control [10]. The experimental carrier shapes of above researches mainly focused on bionic fishes but there are few studies directly on applications of underwater vehicles. 

To promote the application of the artificial lateral-line system in underwater equipment such as underwater vehicles, a cylindrical carrier was utilized as a main research object in this paper. The lateral line sensing mechanism was studied in the perspective of biomechanics, which laid a biological foundation for the design of artificial lateral-line system. The optimal sensor distribution model and underwater environment perception algorithm were obtained through the hydrodynamic simulation. An artificial lateral line system based on the optimal topology of the sensors was designed. The underwater experiments verified the validity of numerical simulation by a comparison between the experimental data and the simulation results. The effective methods of flow velocity estimation, attitude perception and obstacle identification based on the artificial system were put forward from the verification experiments.

## 2. Biomechanical Model of Lateral Line

**Figure 1:**
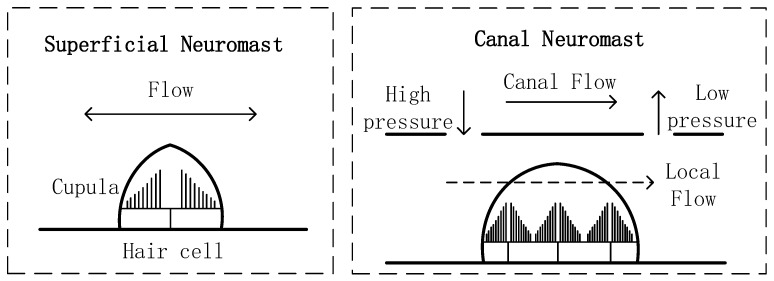
Schematic of Superficial Neuromasts and Canal Neuromasts.

Lateral line is a hydrodynamic receptor system in fishes and amphibians, which mainly senses the local water pressure, vibration and other hydrodynamic stimuli [11]. The organizations of lateral organs are various in different species. Biologists found that the lateral organ was composed of neuromasts, which are distributed on both sides of the running fish body. According to the location, neuromasts are divided into two types, superficial neuromasts (SNs) and canal neuromasts (CNs) [12]. Their biomechanical model is shown in Figure 1. 

Netten et al. established a biomechanical model of lateral sensing neuromasts, which provided the basis for the development of artificial lateral line systems [13]. The quantitative analysis of his frequency response formula facilitated the understanding of the hydrodynamic input and perceived output.

The frequency response of SNs is defined as [14]

(1) $$\;S_{SN}\left(f\right)=\frac{\upsilon \left(H\right)}{U_{\infty }}=-\frac{ib_{w}}{2\pi fb_{m}}\left[1-{\frac{i\pi fb_{m}\delta^{4}}{2EI+i\pi fb_{m}\delta^{4}}}^{\left(\frac{-H\left(1+i\right)}{\delta }\right)}\right]+\overset{3}{\underset{j=0}{\sum }}C_{j}{}^{\left(i^{j}H^{4}\sqrt{\frac{2\pi fib_{m}}{EI}}\right)}$$

where υ(H) is cupular deflection at the height of the beam tip, $U_{\infty }$ is free-stream velocity,

(2) $$b_{m}=-4\pi \mu k-i\pi \left(\rho_{w}+\rho_{m}\right)a^{2}\omega +\frac{i\pi^{2}\mu k\;}{L}\;$$

(3) $$b_{w}=-4\pi \mu k-2i\pi \rho_{w}a^{2}\omega +\frac{i\pi^{2}\mu k}{L}$$

where *μ* is the dynamic viscosity of fluid. *k* is viscous force coefficient, $\rho_{w}$ is the density of water, a is radius of the hemisphere, $C_{j}$ is a length of 4 constant sequence, $\rho_{m}$ is cartilage peak matrix density, *H* is the height of the cartilage peak *L* is defined as:

(4) $$L=\gamma +\ln(\frac{a}{\sqrt{2\delta }})$$

where γ is Euler’s constant (γ≈0.5772), δ is boundary layer thickness.

We focused on the bionics analysis rather than mathematics. SNs are similar to displacement transducers and sensitive to flow velocity because the displacement of the cupula is relative to viscous drag and consequently to the velocity of water particles. So SNs can estimate the flow velocity and orientation on their body surface.

The frequency-dependent sensitivity of CN cupula can be defined as [15]:

(5) $$S_{CN}\left(f\right)=\frac{Y_{0}\left(f\right)}{V_{0}\left(f\right)}=\frac{1}{2\pi f_{t}}\frac{1+\frac{\sqrt{2}}{2}\left(1+i\right){\left(\frac{f}{f_{t}}\right)}^{\frac{1}{2}}+\frac{1}{3}i\frac{f}{f_{t}}}{N_{r}+i\frac{f}{f_{t}}-\frac{\sqrt{2}}{2}\left(1-i\right){\left(\frac{f}{f_{t}}\right)}^{\frac{3}{2}}-\frac{1}{3}{\left(\frac{f}{f_{t}}\right)}^{2}}$$

where $Y_{0}(f)$ is vibration amplitude of cartilage peak amplitude, $V_{0}(f)$ is external flow velocity amplitude, $f_{t}$ is defined as

(6) $$f_{t}=\frac{\mu }{2\pi \rho_{w}a^{2}}$$

where *μ* is the dynamic viscosity of fluid. $\rho_{w}$ is the density of water, *a* is radius of the hemisphere, $f/f_{t}$ is Stokes number, *N_r_* is defined as the resonance factor that governs the resonance properties of a cupula.

CNs are regard as acceleration sensors because the canal fluid velocity is practically proportional to the first full derivative of the flow speed outside the canal [16]. And CNs can also be regard as pressure gradient sensors because the water acceleration is proportional to the pressure gradients. Thus, CNs detect the pressure distributions in their lateral line canals [15]. SNs can distinguish fields in spatially uniform flow and turbulent flow while CNs only respond non-uniform flow fields, such as water fluctuations generated by a vibrating sphere or a swimming fish [17]. The artificial lateral line system combined features of SNs and CNs. Miniature pressure sensors are arranged on the surface of the carrier and the flow field characteristics are obtained by the surrounding pressure field. 

There are three main research goals:

1. Flow velocity estimation
2. Attitude perception
3. Obstacle identification.

All the experiments are carried out in uniform flow or regular non-uniform flow (Karman vortex streets).

## 3. Optimal Topology of Sensors

The artificial lateral line system is equipped with a number of pressure sensors, so the distribution of sensors should be studied. More sensors should be placed on the most sensitive locations [18] of the water flow environment. Imitating an underwater aircraft shape [19,20], the artificial carrier is cylinder with a hemispherical head. Simulation is carried out through ANSYS FLUENT with basic parameters in the Table 1.

**Figure 2:**
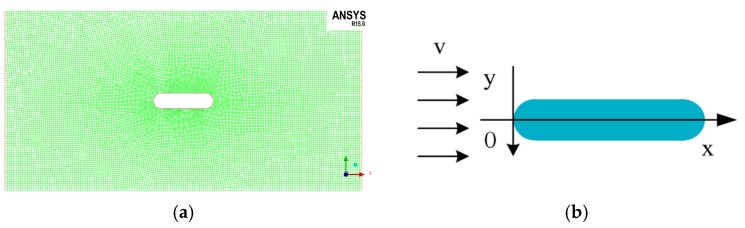
The results of the meshing and the definition of the axes. (**a**) The plane mesh; the green area represents the fluid domain and the oval shape represents the carrier. (**b**) The definition of axes; the dark blue oval shape represents the carrier in the picture.

In the three-dimensional simulation analysis, it can conclude that the static and dynamic pressure value is almost the same in the cross-section perpendicular to the carrier axis, so it can be simplified as two-dimensional flow field simulation. The plane mesh result is Figure 2a. As shown in Figure 2b, the pressure data of the surface of the carrier is extracted along the edge of the section. The zero point is the front end of the carrier, the upper part is the negative direction and the lower part is the positive direction. Starting from zero, the length of the curve along the edge of the section is defined as “carrier surface length.”

**Figure 3:**
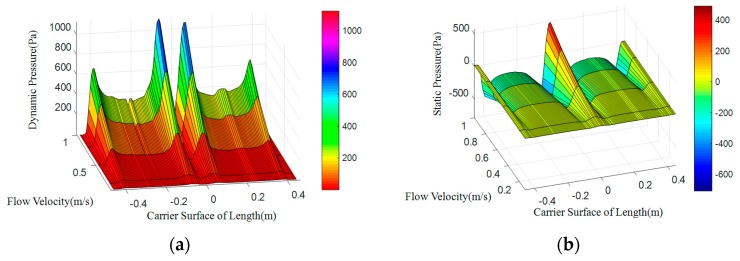
Pressure distribution of carrier surface at different flow velocity. (**a**) Dynamic pressure; (**b**) Static pressure.

Simulation results with flow velocity of 0.1 to 1 m/s calculated by FLUENT are integrated by MATLAB and shown in Figure 3.

As shown in Figure 3 is the relationship between surface pressure, velocity and geometry, that is, the overall pressure distribution model, the specific data can be obtained by picking up surface points. The location of extreme points is shown in Table 2. As the flow velocity increases, the value of surface pressure gradually increases and the position of the extreme point does not change. The pressure slope of maximum point is the highest, thus it is the most sensitive to flow velocity and suitable for pressure sensors placement. By observing the characteristics of pressure changes, we can see that sensors are deployed at the mid-point of the pressure distribution and at the maximum point and the minimum point of the pressure data, which can detect the pressure changes more sensitively, so that the position is suitable for arranging the sensor. In addition, the pressure change at the zero point of the static pressure is 0, which can be used as the pressure reference point. Therefore, the pressure sensor is also suitable here. 

According to the variation of static pressure between different flow rates, it can be seen that the static pressure keeps zero near X=±0.03, because the initial pressure of velocity inlet and pressure outlet is set to 0 under simulation conditions, so it can be used as the basis for judging the current depth of carrier. Due to the maximum point of hydrodynamic data and the minimum point of hydrostatic data are rather special and the coordinate position needs to be determined according to the calculation. Because of the symmetrical distribution on the left and right, first take the average of the abscissa as follows:

(7) $$\bar{x_{1}}=\left(0.075+0.066+0.057+0.066\right)/4=0.066\;m$$

(8) $$\theta_{1}=\bar{x_{1}}/r=0.066/0.05=1.32\;\mathrm{rad}=75.67^{\circ }$$

(9) $$\bar{x_{2}}=\;[\left(0.914/2-0.387)+(0.914/2-0.384)\right]/2=0.0715\;m$$

(10) $$\theta_{2}=\bar{x_{2}}/r=0.0715/0.05=1.43\;\mathrm{rad}=81.97^{\circ }$$

(11) $$\bar{x_{3}}=\left(0.03\;+\;0.03\right)/2=0.03\;m$$

(12) $$\theta_{3}=\bar{x_{3}}/r=0.03/0.05\;=\;0.6\;\mathrm{rad}=34.4^{\circ }$$

**Figure 4:**
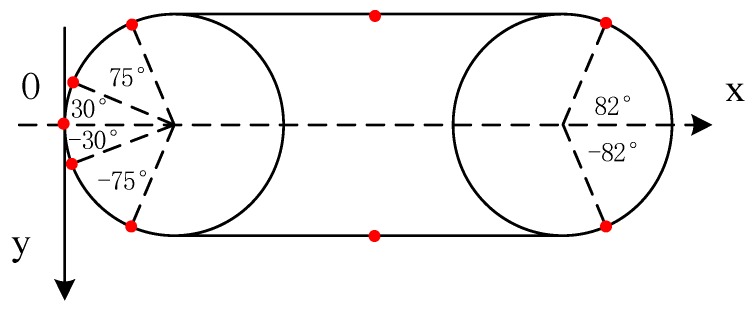
The pressure sensitive point on carrier surface.

According to the geometric relationship between the surface length and diameters of hemispherical head, the position of the feature point can be obtained, shown in Figure 4.

**Figure 5:**
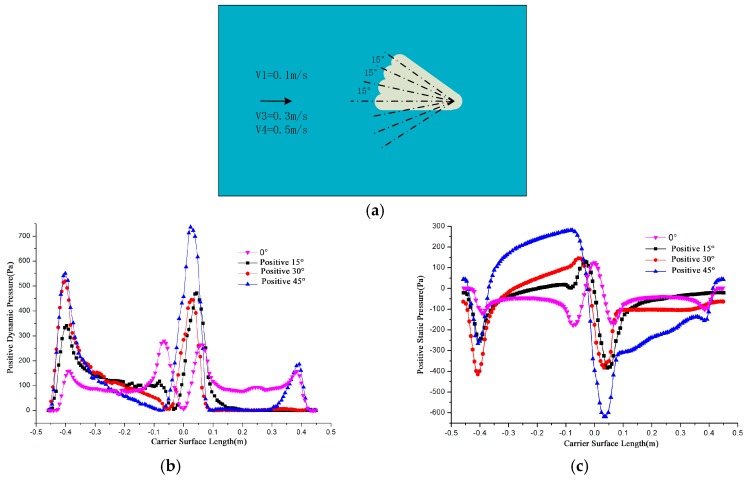
The surface pressure curve in different angles under 0.5 m/s. (**a**) A schematic of angle; (**b**) Dynamic pressure; (**c**) Static pressure.

When the carrier and the flow direction are 15°, 30°, 45° respectively and flow velocity is fixed at 0.5 m/s, the surface pressure distribution is shown in Figure 5. Due to the vortex at this time and the surface pressure is related to vortex shedding frequency, the simulation time is fixed at 2.5 s.

Figures indicate that the cylindrical area pressure is almost unchanged in symmetry distribution when the carrier is facing the flow (0°). As the angle increases, the symmetry is broken, the dynamic pressure of incident flow surface (y < 0) increases the countercurrent surface pressure decreases, the stagnation point moves, the original two maximum points reduced to one.

The arrangement of the sensor spacing in the cylinder area can be calculated from pressure slope of incoming flow surface and the sensitivity of pressure sensor. Assume that the sensor sensitivity is *S_p_*, the sensor spacing is x and the pressure slope is $k_{p}$. x can be expressed as:

(13) $$x=\frac{s_{p}}{k_{p}}$$

The pressure gradients that the system can capture are proportional to the sensor spacing, so there is a minimum distance between the two sensors; at that time the pressure difference between two sensors is exactly the sensor resolution. Assume that the sensitivity to the angle is 15°, simulation showing the dynamic pressure slope on incoming flow surface is 281 Pa/m. The sensor type of artificial lateral line is MS5803-07BA, which possess resolution of 0.097 mbar with the digital-analog conversion sampling frequency at 512 Hz. The minimum distance $X_{\min}$ can be written as:

(14) $$X_{\min}=\frac{S_{p}}{k_{p}}=\frac{9.7}{281}=0.035\;m$$

**Figure 6:**
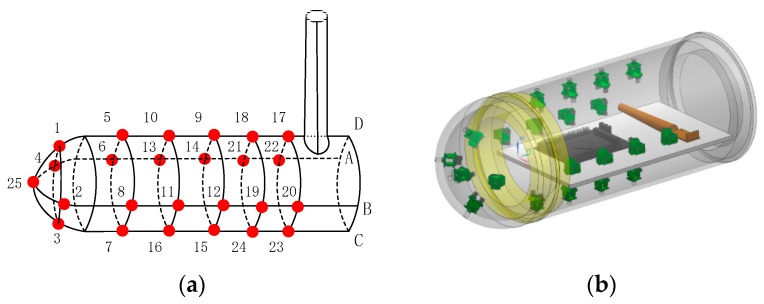
Artificial lateral line system. (**a**) Sensor distribution; (**b**) 3D modeling. This model includes the shell, sensors and embedded hardware.

Considering that the artificial lateral line system produces the process and circuit arrangement, the sensors are spaced with 50 mm between each other. Five columns are arranged in the cylinder surface, shown as Figure 6.

In this paper, we obtain the pressure distribution cloud of the carrier by hydrodynamic simulation of the carrier and find the pressure sensitive location of the carrier from the pressure cloud graph, such as the pressure maximum point, the pressure minimum point and the static pressure zero point. These locations are the best locations for sensor placement. No matter how the shape and size of the carrier changes, it can be analyzed in this way to find the best location for sensor placement.

## 4. Obstacle Sensing Algorithm Based on Simulation

Turbulence fluid environment is complex in irregular vortex or gradient flow. In order to obtain an obstacle identification method, a periodic fluid environment formed by Karman vortex streets behind an obstacle is utilized as a simulated scene to represent turbulent flow. This environment can be reproduced by the experimental sink, which can easily verify the simulation results through the experimental data. It can be assumed that the all faced with an obstruction in front of it under certain flow velocity. Its surface pressure distribution will differ from the shape, dimension and motion of the obstacles, which can be features to obtain sensing algorithm.

### 4.1. Simulation of Static Obstacle

**Figure 7:**
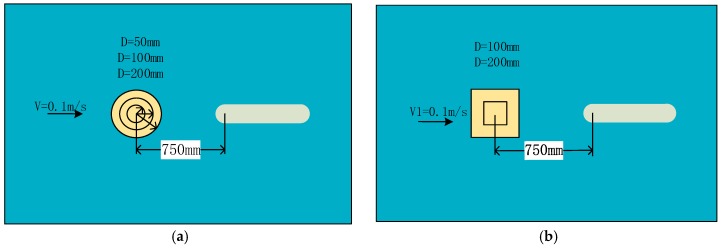
Simulation of static obstacle. (**a**) Cylindrical obstructions; (**b**) Square obstructions. The circle represents a circular obstacle, the square represents a square obstacle, and the oval shape represents a carrier.

**Figure 8:**
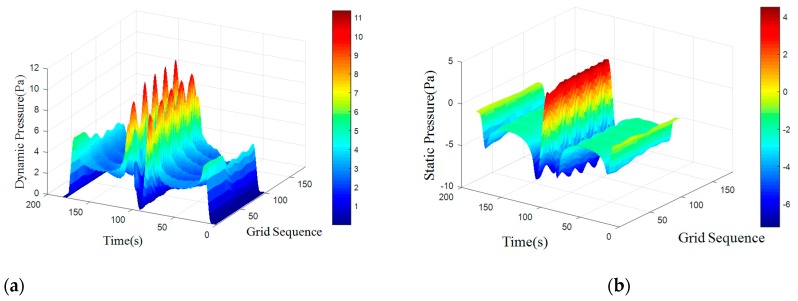
Cylindrical obstructions with diameter of 50 mm. (**a**) Dynamic pressure; (**b**) Static pressure.

**Figure 9:**
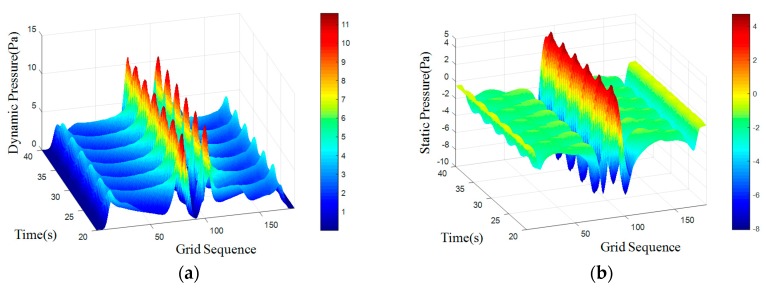
Cylindrical obstructions with diameter of 100 mm. (**a**) Dynamic pressure; (**b**) Static pressure.

**Figure 10:**
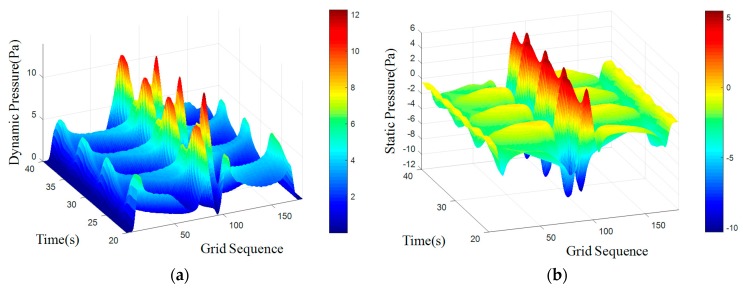
Cylindrical obstructions with diameter of 200 mm. (**a**) Dynamic Pressure; (**b**) Static Pressure.

**Figure 11:**
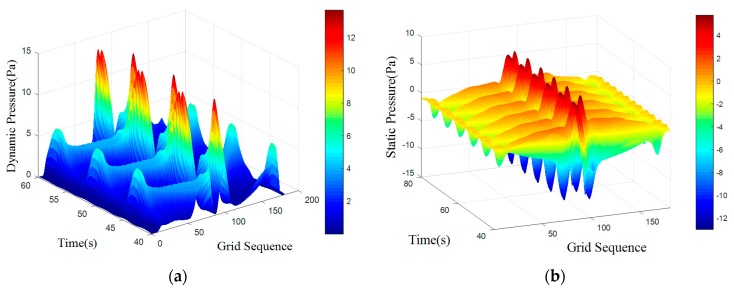
Square obstructions with side length of 100 mm. (**a**) Dynamic Pressure; (**b**) Static Pressure.

The simulation settings are shown in Figure 7. Only one obstacle was located in the flow field during each simulation. When flow passed through a certain object under some kind of fluid environment, a column of regular vortex will be formed behind the object called Karman vortex streets. The vortex shedding frequency calculation formula is as follows:

(15) $$S_{t}=\frac{fd}{V}=0.198\left(1-\frac{19.7}{Re}\right)$$

where *S_t_* is for the Strauss number, it is maintained at 0.21 when Reynolds number is in the range of 300 to 10^5^. A regular Karman vortex streets occurred when Reynolds number in the range of 60–10^5^. The simulated surface pressure results are shown in Figure 8, Figure 9, Figure 10 and Figure 11. The *X*-axis is a grid node sequence distributed along the surface of the carrier, with a total of 182 nodes, the 91st grid node is the forefront of the head; the *Y*-axis is a time series. According to the simulation results, 10–20 s, 20–40 s, 30–60 s range; *Z*-axis pressure value.

The figures showed that at a certain grid node, all pressure curves were superposition of a direct current component with a periodic component, which conformed to a second order Fourier polynomial. In the periodic component, the period of dynamic and static pressure under same obstacle was similar. By means of de-mean fast Fourier transform, the frequency domain was obtained. There were three main frequency peaks. In the same simulated environment, the amplitude of once frequency at stagnation point was the highest, the second and the third frequency amplitude gradually decreased. While the dimension of the obstacle increased, all the frequency amplitude increased. Taking once main frequency as a simulated characteristic frequency, the comparison for the theoretical frequency of Karman Vortex shedding to the simulated frequency is shown in the Table 3. The feature size of a square obstacle is the diameter of its circumcircle. When the length of square obstacle was 200 mm, the carrier entered in the obstacle vortex separation zone and prevented the vortex shedding. So, a stable flow field was formed with no characteristic frequency in pressure.

**Figure 12:**
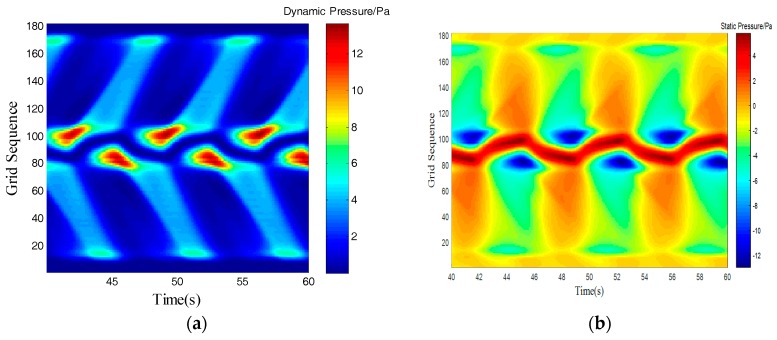
Square obstructions with side length of 100 mm. (**a**) Dynamic Pressure; (**b**) Static Pressure.

**Figure 13:**
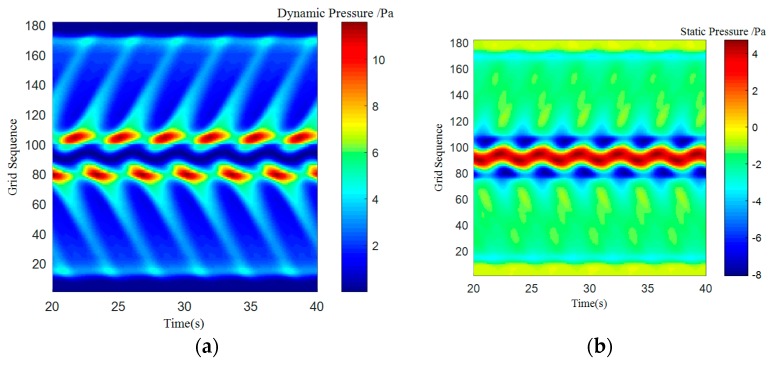
Cylindrical obstructions with diameter of 100 mm. (**a**) Dynamic Pressure; (**b**) Static Pressure.

Table 3 showed that an obstacle in the uniform flow field caused an obvious frequency characteristics in surface pressure distribution and the first main frequency of an artificial lateral line system was closed to the Karman Vortex shedding frequency, which can help estimate the obstacle feature. The surface pressure waveform could distinguish shape of obstacle as Figure 12 and Figure 13 shown.

In the dynamic pressure of circular obstacle, the peak pressure interval is relatively short and the peak pressure is relatively high but the peak interval time of the square obstacle is relatively long. It can be seen from the analysis of static pressure cloud chart that the pressure value of square obstacle in the straight transitional zone is larger and there are more red areas in the cloud image. However, in the pressure cloud chart of the circular obstacle, there are more green areas and the pressure values is smaller.

### 4.2. Simulation of Moving Carrier

**Figure 14:**
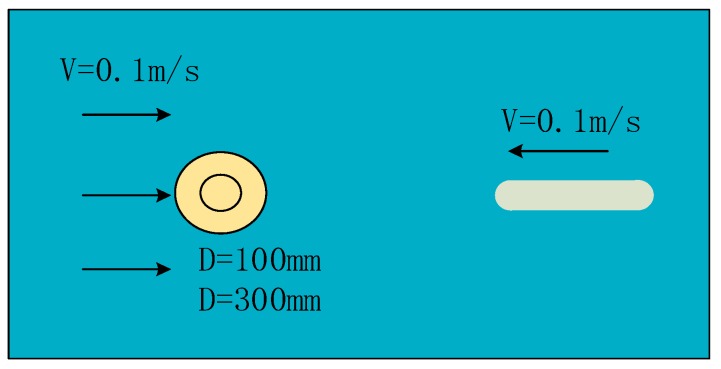
A schematic of the simulation environment.

In this simulation, the carrier gradually moves to an obstacle along the flow direction through dynamic mesh technology in FLUENT. The basic simulated parameter of the environment is shown in Figure 14.

**Figure 15:**
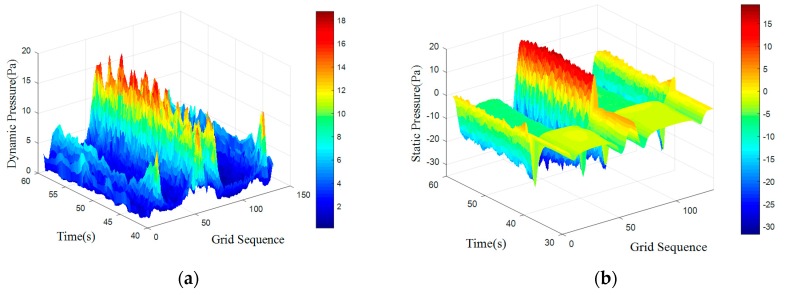
Surface pressure distribution with moving carrier with d = 100 mm. (**a**) Dynamic Pressure; (**b**) Static Pressure.

**Figure 16:**
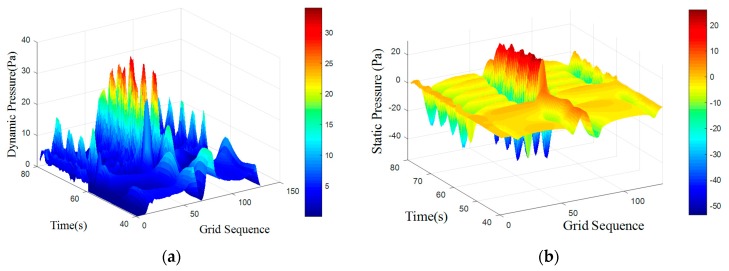
Surface pressure distribution with moving carrier with d = 300 mm. (**a**) Dynamic Pressure; (**b**) Static Pressure.

An UDF was programmed to define the motion characteristics. Smoothing and re-meshing methods were combined to achieve dynamic mesh of the moving carrier. The spring constant factor was set to 0.6, sizing function was open and re-meshing methods were applied to Local Cell, Local Face and Region Face. The surface pressure data simulations form is shown in Figure 15 and Figure 16.

When the carrier moved, the overall trend of the pressure distribution was unchanged. There were still period features on surface caused by obstacles. Table 4 shows frequency domain features through mean-value Fourier transform. There were still three main frequency peaks but the amplitude of first frequency was not the highest. The highest amplitude was underline which represented the characteristic frequency.

Compared to the static state, the characteristic frequency of the motion state was increasing. Assume that the static frequency was $f_{1}$, increasing part was $f_{2}$, moving frequency is *f*′. Carrier moved at speed of 0.1 m/s, the length of it was 0.4 m. There are:

(16) $$f^{\prime }=\frac{v_{1}+v_{2}}{s}=\frac{\frac{s}{T_{1}}+\frac{s}{T_{2}}}{s}=\frac{1}{T_{1}}+\frac{1}{T_{2}}=f_{1}+f_{2}=f_{1}+\frac{0.1}{0.4}=f_{1}+0.25$$

So, the frequency increase under 0.1 m/s was 0.25 Hz. According to Equation (16), the moving frequency in theoretical calculation and simulation can be calculated as Table 5.

Table 5 indicated that the moving frequency was close to the theoretical frequency. The simulation shows that movement of the artificial lateral line would change the frequency distribution of surface pressure. The amount of change was depended on the velocity and shape. Therefore, when identifying an obstacle, the movement of artificial lateral line itself should be considered.

### 4.3. Simulation of Vibrating Obstacle

**Figure 17:**
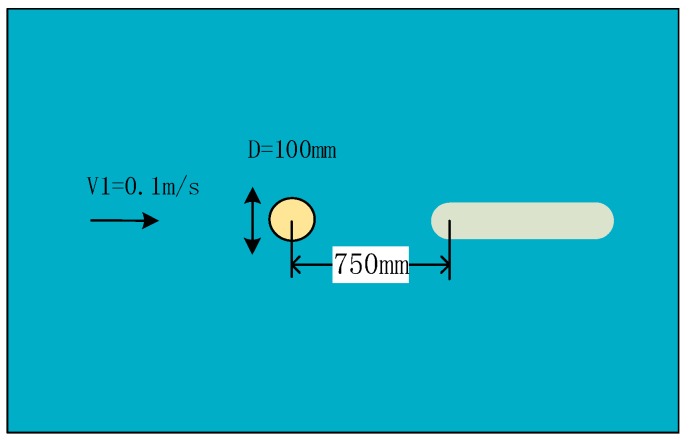
The schematic of simulation environment. In the picture, a circle represents a circular obstacle, and the oval represents a carrier.

The simulation environment was shown in the Figure 17. The carrier was static while the obstacle vibrated. In the static state, the theoretical vortex shedding frequency is 0.21. The FLUENT UDF (User Defined Function) function is used to control the obstacle vibration. The amplitude of the vibration was 0.01 m and the frequency was 0.2 Hz. The vibration equation was as follows:

(17) y=0.01 × sin2π × 0.2t

**Figure 18:**
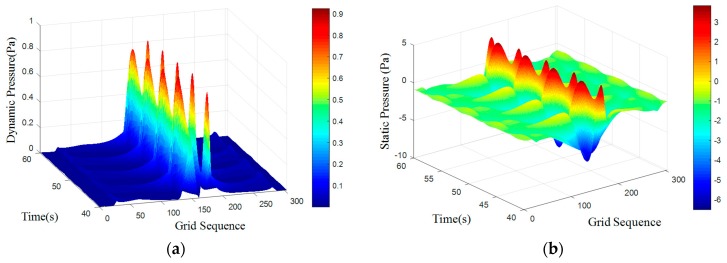
Surface pressure distribution with static carrier. (**a**) Dynamic Pressure; (**b**) Static Pressure.

The result of simulation is shown in Figure 18.

The figures indicated that the Karman vortex phenomenon still existed when the obstacle was slightly vibrating but the back pressure extremes disappeared. Because vibration direction is perpendicular to the flow, the shedding vortexes possessed a velocity component in the direction of vibration which weakened the vortex force when they moved to the end of the carrier. From the frequency domain, the characteristic frequency was 0.199 Hz which was less than it at static state. One of the reasons may be that the vibration reduced the frequency of vortex shedding.

When the amplitude of vibrating increased, the surface pressure waveform became distortion, no longer conformed to a standard second order Fourier polynomial but the main frequency of the pressure was still 0.199 Hz. High-order components appeared in the frequency domain when vibrating frequency increased, which are shown in Table 6.

From the qualitative analysis, the vibration state of the obstacle can be identified by the extreme value of the back pressure.

In the simulation analysis of two-dimensional flow field, the movement of the carrier leads to the increase of the frequency characteristics of the pressure signal on the surface, which is positively related to the velocity. The Karman vortex phenomenon still exists in the small amplitude vibration of the obstacle but the extreme value at the tail of the dynamic pressure disappears and the value of dynamic pressure is much smaller than that of the stationary obstacle. The vibration reduces the frequency of vortex shedding, which leads to the decrease of the characteristic frequency of pressure on the surface of the carrier. The vibration amplitude affects the waveform of pressure and the vibration frequency leads to high-order components in the pressure frequency domain. The square obstacle is similar to a circle; however, it is necessary to use the diameter of the circumscribed circle to calculate the shedding frequency of Karman vortex shedding. By obtaining the influence of the change of the parameters on the experimental results, it can provide the basis for the establishment of the algorithm of identifying the shape, the size and the movement state of the obstacle. In the experiment, we set the obstacle and carrier distance as 720 mm and carrier shape as torpedo type. In the following experiment, we will further study the influence of these two variables on the perception of flow field.

**Figure 19:**
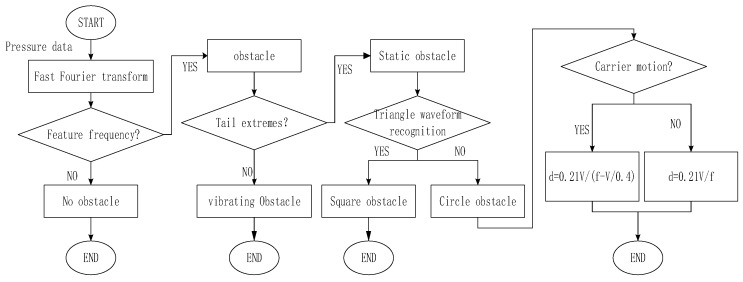
The process of environmental perception method.

A general process of environmental perception method by artificial lateral line was conducted through above analysis, which is shown as Figure 19.

## 5. Experiments of Artificial Lateral Line

There were totally 25 pressure sensors on artificial lateral line surface, which distributed as figure. Microcontrollers, wireless communication modules and batteries were placed inside the carrier while charging and powering were outside through two watertight connectors. The whole system was sealed by O-ring.

### 5.1. Experiments Design

**Figure 20:**
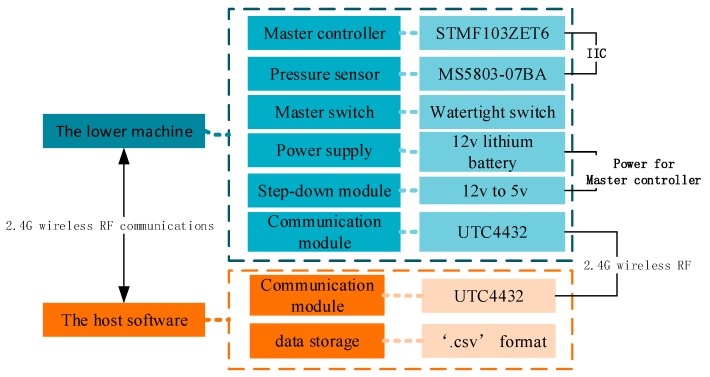
The overall program about control system.

The overall control system was divided into the host and the lower machine, the lower machine was running in underwater carrier, the host was a data received software running on computers, the overall program is shown in Figure 20.

**Figure 21:**
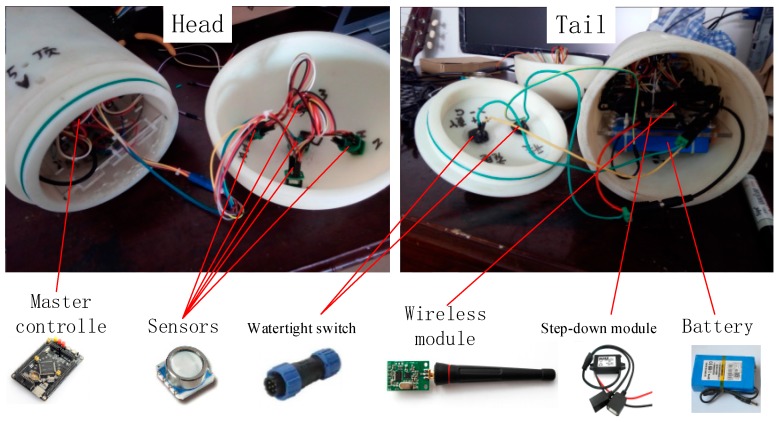
The physical hardware connection of lateral line.

There were totally 25 pressure sensors which were numbered according to the Figure 6. The supply voltage of sensors is 3.3 V, which is given by the voltage output of mater controller. The sensor resolution is 0.04 mbr when the oversampling ratio is 4096 Hz, meanwhile the analog-to-digital (DA) conversion response time is 8.2 ms. Limited to the DA conversion time of the digital sensor, the pressure signal sampling frequency of artificial lateral line system was 9.524 Hz. The host software programmed in C# language and could receive and store pressure data in real time. The physical hardware connection is shown in Figure 21.

### 5.2. Underwater Experiments

This experiment was carried out in an experimental tank. One end of the tank is equipped with a high-power pump for generating a flow. The specific parameters of the experimental sink are shown in Table 7.

**Figure 22:**
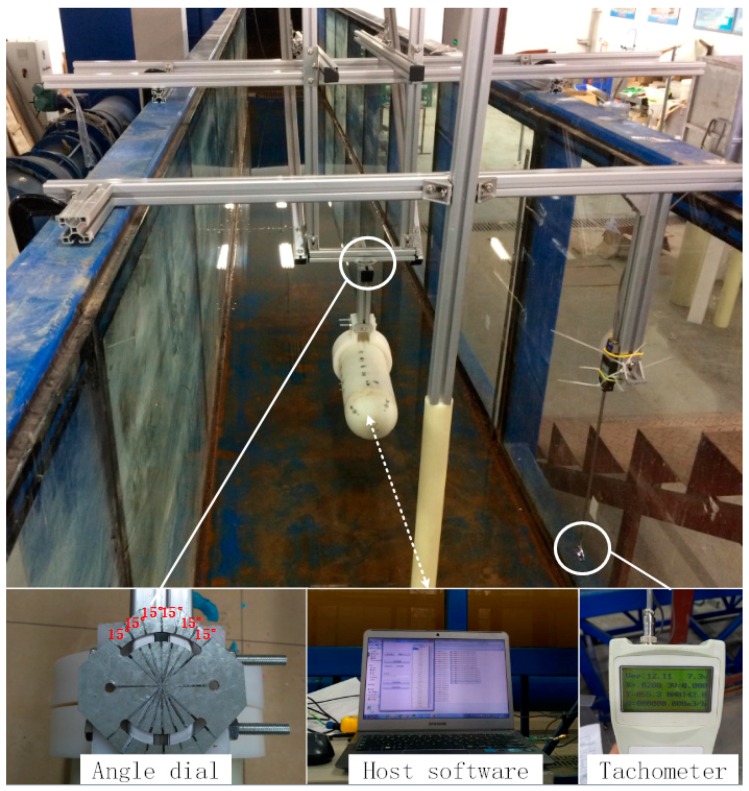
The sink experiment of lateral line.

Artificial lateral line was placed in the middle of the tank with wireless communication device inside. In order to ensure the integrity of wireless signal, the artificial system was installed underwater for 20 cm to 30 cm. The experiment was shown in Figure 22.

In order to collect static characteristic as a control group, the artificial carrier was placed in still water and recorded pressure data before generating a flow. The static characteristic was related to the current depth, water temperature, inherent characteristics of the sensors and other environmental factors. The experimental data need to be processed after removing the static characteristics. The experiments were conducted according to research goals in Section 2 and simulation parameters.

## 6. Experimental Analysis of Artificial Lateral Line 

Experimental data could verify simulation results and obtain the method of underwater fluid detection. Due to improper installation, sensors 7, 10, 12, 16 and 18 were unrecoverable mechanical damage, there remain 20 available sensors.

### 6.1. Hydrostatic Correction

**Figure 23:**
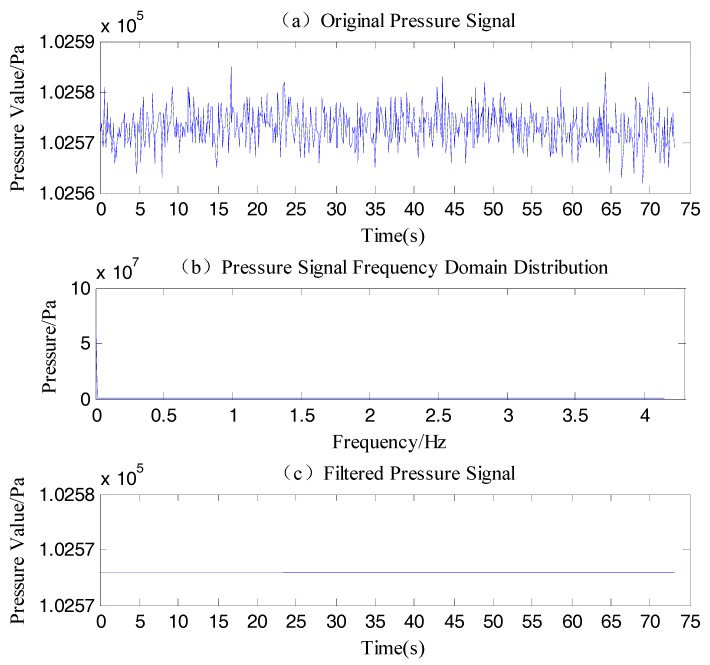
The process of the No. 1 sensor pressure data.

The pressure data of the No. 1 sensor was shown in Figure 23a, the frequency domain obtained by fast Fourier transform was shown in Figure 23b. There was an obvious static characteristic in the original signal, because the highest amplitude appeared at 0 Hz and other high frequency components was 0 Pa. Through low-pass filtering to remain only 0 Hz, the pressure data was converted to time domain by inverse Fourier transform. Figure 23c was the hydrostatic pressure after the processing, which was averaged. The hydrostatic pressure of all underwater sensors was obtained by this method. 

### 6.2. Velocity Estimation

The experimental data subtracted hydrostatic pressure was compared with the static pressure in the simulation. For uniform flow field, there were three methods, respectively, stagnation pressure fitting, static pressure fitting, Bernoulli method, for turbulent flow field, there was Karman vortex method.

Stagnation pressure fitting method:

When the carrier was facing the flow, the center of the head was stagnation point (point A in simulation, sensor 25 on carrier). According to the simulation results, the relationship between pressure and flow velocity at point A was as follows:

(18) $$P_{s}=485.9v^{2}+5.033v-0.41$$

According to the experimental data, the actual fitting curve is:

(19) $$P_{t}=172.2v^{2}+135.75v-17.55$$

The fitting degree between the simulation curve and the experimental data was 0.9675, while the actual fit degree was 0.9755.

**Figure 24:**
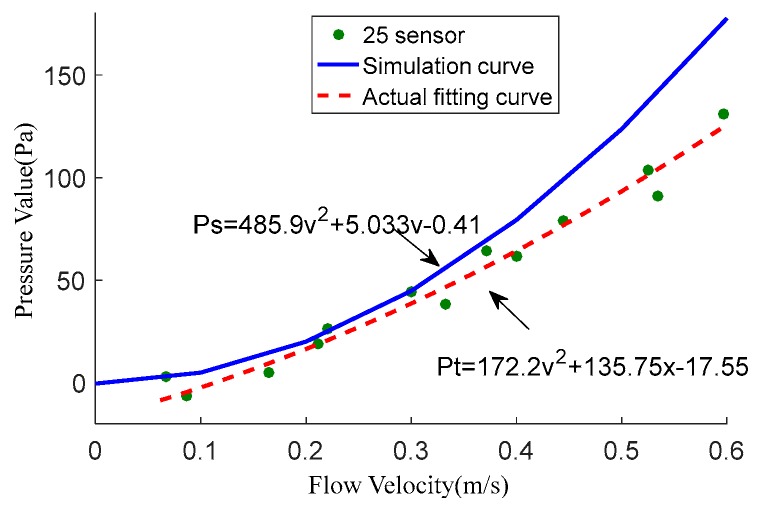
The results fit of the simulation.

Figure 24 showed that the simulation results fit well to experimental data under low velocity, while the simulation was higher than the actual data in the case of high velocity. Since the simulated fluid was set to ideal liquid water, the experimental water was turbid. The density and viscosity of the ideal fluid are all constant. But for turbid water, these are all changing. As the flow rate increases, the water quality becomes more turbid, the internal friction in water increases and the dynamic viscosity of water increases. As a result, the loss along the water flow increases and the pressure loss getting bigger and bigger. But this situation is not in the simulation, so the simulation was higher than the actual data in the case of high velocity. At the same time, experimental instruments such as the gun, the sensor itself is also present measurement error. According to the cumulative error of experimental instruments, as the flow rate increases, the error will also increase. This is also why the simulation data is higher than the experimental data.

**Figure 25:**
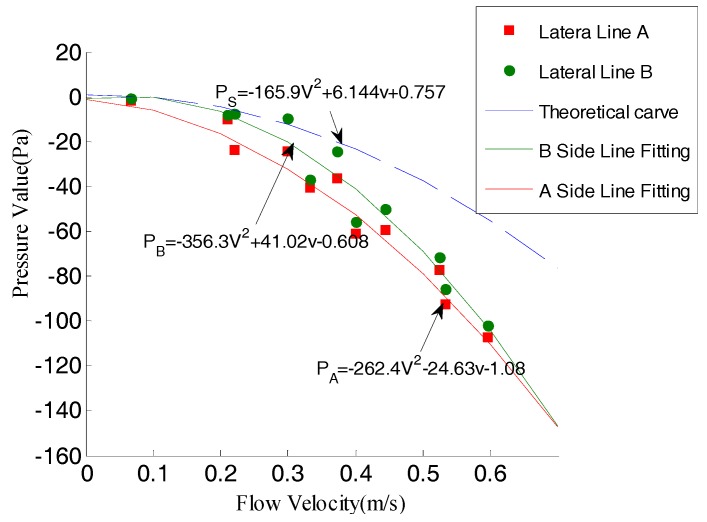
The pressure curve fitting.

Static pressure fitting method:

Take sensors 9, 14, 21, 22 to represent the lateral line A and sensors 8, 11, 19, 20 to represent the lateral line B. The corresponding location in simulation was point C (see Figure 25). The theoretical curve was fitting curve of point C, which could be expressed as:

(20) $$P_{s}=-165.9v^{2}+6.144v-0.7572$$

Average value of each lateral line sensor was their mean static pressure. The fitting degree between theoretical curve and experimental data were 0.9657 (lateral line *A*) and 0.9476 (lateral line *B*). According to sensor data, the actual fitting equations were:

(21) $$P_{A}=-262.4v^{2}-24.63v-1.08$$

(22) $$P_{B}=-365.3v^{2}+41.02v-0.608$$

The actual fitting degree were 0.9673 (lateral line *A*) and 0.9486 (lateral line *B*).

Bernoulli method:

According to Bernoulli’s law, the fluid’s stagnation pressure is equal to the sum of static pressure and dynamic pressure, which can be written as:

(23) p0=pfs+ρV22 →Vt=2(p0−pfs)ρ

where $p_{0}$ represents stagnation pressure, $p_{fs}$ represents static pressure, *ρ* is fluid density and $V_{t}$ is the theoretical velocity. In the experiment, sensor No. 25 collected stagnation pressure and the average value taken by lateral line A and B was static pressure. It was assumed that $V_{e}$ representing actual flow velocity measured by the flow meter. Because of the system error, there should be a correction factor *β* as follows:

(24) $$\beta =\frac{V_{e}}{V_{t}}=\frac{V_{e}}{\sqrt{2\left(p_{0}-p_{fs}\right)/\rho }}$$

The fitting degree between *V_e_* and *V_t_* was 0.9925.

**Figure 26:**
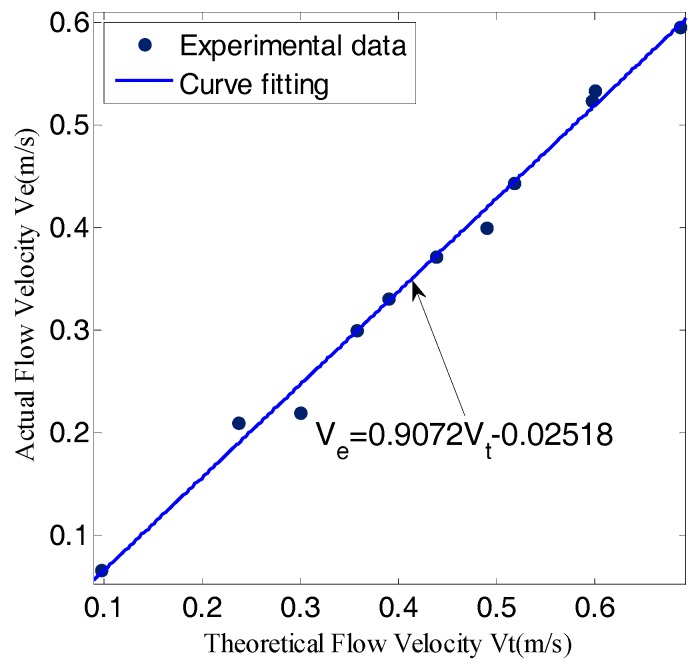
The curve fitting of the experimental data.

Figure 26 showed that *β* was 0.9072, so the actual flow velocity can be expressed as:

(25) $$\begin{array}{ll} V_{e} & =0.9072V_{t}-0.02518=0.9072\sqrt{2\left(p_{25}-\bar{p_{ABCD}}\right)/1000}-0.02518\\ & =0.0405\sqrt{\left(p_{25}-\bar{p_{ABCD}}\right)}-0.02518 \end{array}$$

where $p_{25}$ is the pressure of sensor No. 25, $\bar{p_{ABCD}}$ is the average value taken by lateral line A and B was static pressure.

Karman vortex method:

For turbulent field, the obstacle diameter was fixed at 100 mm. As the velocity increased, the Karman vortex appeared clearly. Due to the complexity of the fluid environment, the current velocity cannot be obtained by the Bernoulli method. Due to the formula of Karman vortex shedding frequency, in case of known obstacle dimension, the current flow velocity can be calculated according to the characteristic frequency of the pressure signal.

**Figure 27:**
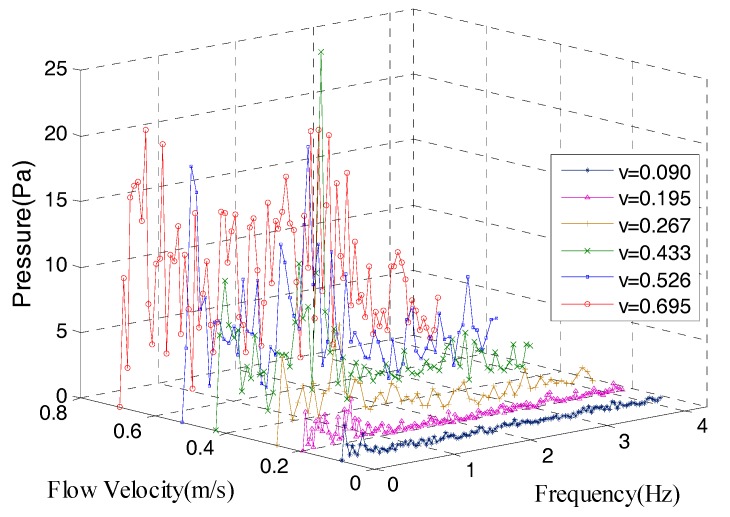
The amplitude-frequency characteristic of the sensor No. 2.

The amplitude-frequency characteristic of the sensor No. 2 shown in Figure 27 was obtained by fast Fourier transform. It can be seen from the figure that with the increase of the flow velocity, the average amplitude of the data increased and the higher amplitude of frequency appeared. According to the Karman vortex formula, the relationship between flow velocity and the frequency when the obstacle diameter is 100 mm was:

(26) $$S_{t}=\frac{fd}{V}=\frac{f\times 0.1}{V}=0.21\to V=0.476f$$

For the actual pressure signal, there is more extreme pressure among different frequency ranges. Taking the high frequency pressure *p_max_* as a criterion, when *p*(*f*) > *p*_max_, the corresponding frequency was regarded as the characteristic frequency, *p*_max_ was written as:

(27) $$p_{\max}=\bar{p}+1.5\sigma$$

The actual fitting relationship was as follows:

(28) V=0.26f+0.067

The fitting effect of each method is summarized in Table 8.

### 6.3. Attitude Perception

**Figure 28:**
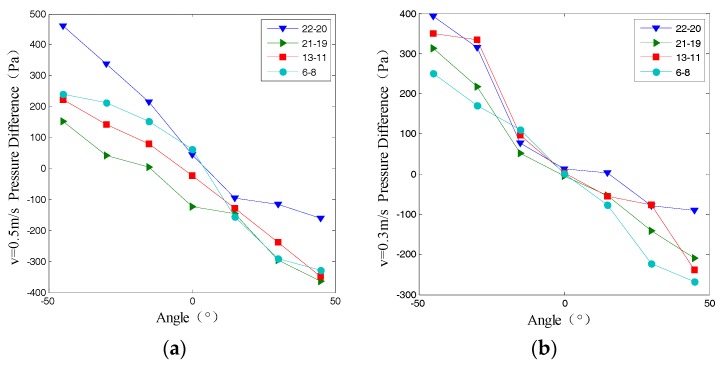
The results of the simulation. (**a**) Pressure Difference with *v* = 0.5 m/s; (**b**) Pressure Difference with *v* = 0.3 m/s.

According to Figure 28, it can be seen that the angle between flow direction and artificial carrier would produce incident flow surface and countercurrent surface, resulting in the pressure distribution difference, which can be foundation to estimate the lateral line attitude. Taking the difference of lateral line A, B corresponding position (6–8, 13–11, 21–19, 22–20) to remove the impact caused by turbulence, pressure difference linear fitting degree was shown in Table 9.

From the Table 9 we can see that the average fitting degree of 0.9 or more, it indicated that the angle and the pressure difference were in a linear relationship, which was as follows:

(29) $$\{\begin{array}{c} P=-3.3A+17.55\left(V=0.1\right)\\ P=-5.9A+42.35\left(V=0.3\right)\\ P=-6.6A-15.66\left(V=0.5\right) \end{array}$$

where *P* is the pressure difference, *A* is the angle between flow direction and artificial carrier.

### 6.4. Obstacle Identification

When there was a significant peak in the frequency domain, it could be determined that there were obstacles in flow field. In case of known flow velocity, the Karman vortex shedding formula can estimate obstacle size with characteristic frequency. According to simulation results, the feature size of square obstacle was its circumscribed circle diameter. The summary data were shown in Table 10. The estimated value was generally smaller than the actual obstacle size, the average error rate was about 22.72%. 

**Figure 29:**
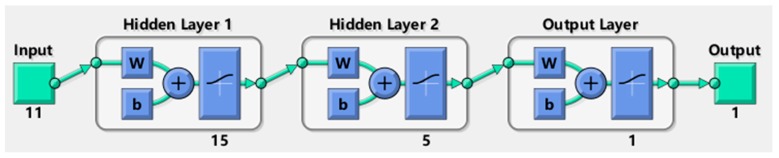
The network topology. In the picture, green indicates the input and output layers, purple indicates the hidden layer, w is the weight, b is the offset, and ∼ indicates the activation function.

The neural network algorithm was used to estimate the shape of the obstacle in a turbulent environment. The output of the circular obstacle was set to 0, while the square obstacle was 1. The experimental data was input in trained model to identify the obstacle shape. Take k1 = 15, k2 = 5. The transfer function between the three layers uses the logarithmic S-type transfer function, the training function is based on the train-Berger-Marquardt reverse propagation method, the learning function uses the BP learning rule, Performance analysis function uses mean square error performance analysis, network topology is shown in Figure 29. 

**Figure 30:**
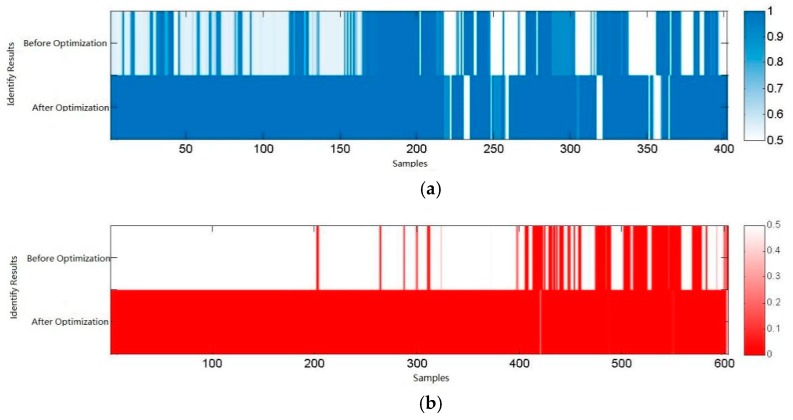
The output of network. (**a**) Square obstacle identification; (**b**) Circle obstacle identification.

After the optimized training model, the recognition rate of square obstacle improved to 95.3%, the recognition rate of circular obstacle was 98.9%. The output of network is shown in Figure 30. 

## 7. Conclusions

In this paper, a cylindrical artificial lateral-line carrier combined with biomechanics, bionics, fluid mechanics and embedded system is developed. Firstly, by imitating the mechanism of fish lateral canal neuromasts, a program of artificial lateral-line system embraced with pressure sensors is put forward. Through the hydrodynamic simulation, the mathematical relationship between pressure, flow velocity and shape are obtained and the optimal distribution of the lateral pressure sensor is concluded, the lateral line pressure distribution models in a uniform and turbulent flow field are established.

Then, the artificial lateral line system and the corresponding carrier are designed and realized based on the optimal topology of the sensor. The real-time communication and data transmission between the carrier and the computer processing terminal are realized by using the wireless data transmission module. On this basis, the construction of the artificial lateral line system flow field perception of underwater test platform is realized.

Finally, the validity of the numerical simulation method is verified by the comparison between the experimental data and the simulation results. The effective methods of flow velocity estimation, attitude perception and obstacle identification based on the artificial lateral line are formed, which lays a theoretical foundation for the underwater environment perception.

In this research, the concept of coupled bionics is introduced into the underwater sensing system creatively. It is proposed that the fish lateral line system should be applied to underwater environment perception and navigation, breaking the traditional detected ways based on acoustic, optical and inertial navigation. The method of bionics and motion control is extended to form the knowledge system of underwater vehicle omnidirectional perception and local precise navigation and positioning, which provides the method and technical basis for the application of artificial lateral line system in the field of underwater vehicle.

## Figures and Tables

**Table 1:** The basic parameters of simulation.

**Mesh (Icem)**			
Fluid Dimensions	1 m × 3 m	Carrier Dimensions	0.1 m × 0.4 m
Number of Grids	232,776	Grid type	Unstructured grids
**Hydrodynamic Simulation (Fluent)**			
Physical model	Standard K-ε model	Boundary conditions	Velocity inlet/pressure outlet
Inlet velocity	−1 m/s	Reynolds number	49,900–499,000

**Table 2:** The location of extreme points.

Extreme Points	Dynamic Pressure	Static Pressure
Maximum point coordinates	−0.387	−0.456 0 0.456
	−0.066	
	0.057	
	0.384	
Minimum point coordinates	−0.456 0 0.456	−0.387
		−0.066
		0.057
		0.384

**Table 3:** Comparison of the theoretical frequency of Karman Vortex shedding with the simulated frequency.

Obstacle Dimensions	Round				Square
Feature size/mm	50	100	200	300	100 (141)
Theoretical frequency/Hz	0.42	0.21	0.105	0.07	0.141
Simulation frequency/Hz	0.4918	0.298	0.165	0.096	0.149

**Table 4:** The frequency domain features.

Diameter/mm	Main Frequency Peak/Hz		
100	0.05	0.201	0.452
300	0.05	0.256	0.513

**Table 5:** The comparison of theoretical and simulation results when the carrier moving.

Diameter/mm	Theoretical Shedding Frequency/Hz		Simulation Frequency/Hz		Moving Simulation Frequency/Hz
	Static	Moving	Static	Moving	
100	0.21	0.46	0.298	0.548	0.452
300	0.07	0.32	0.096	0.346	0.256

**Table 6:** The change of frequency when vibrating frequency increased.

Vibrating FrequencyHz	Pressure Main FrequencyHz			
0.2	0.049	0.199	0.298	0.398
0.4	0.049	0.149	0.248	0.348
0.6	0.149	0.248	0.348	0.447

**Table 7:** The specific parameters of the experimental sink.

Item	Parameters
Pool size	1 W × 1.14 (H)(m)
Water density	1.0 × 10^3^ kg/m^3^
Experimental water temperature	18 °C
Maximum flow rate	0.8 m^3^/s
Maximum ideal flow velocity	0.8 m/s

**Table 8:** The fitting degree of each method.

Flow Field	Velocity Estimated Method	Fit Degree
Uniform	stagnation pressure fitting	0.9755
	static pressure fitting	0.94–0.96
	Bernoulli method	0.9925
turbulent	Karman vortex method	0.9893

**Table 9:** Pressure difference linear fit degree.

Sensor Pair	Fitness	
	*V* = 0.3 m/s	*V* = 0.5 m/s
6–8	0.9856	0.9917
13–11	0.9282	0.9336
21–19	0.9643	0.9811
22–20	0.8534	0.9538
Mean	0.9328	0.965

**Table 10:** The summary data of simulation results.

Characteristic Frequency (Hz)	Velocity (m/s)	Calculated Size (mm)	Actual Size (mm)	Error Rate
0.667	0.482	151.7	D200	24.15%
1.361	0.433	66.8	D100	33.2%
2.140	0.413	40.5	D50	19%
0.477	0.534	235.1	A200 (282.8)	16.86%
0.918	0.492	112.5	A100 (141.4)	20.43%
